# Refractive errors in Tianjin youth aged 6–18 years: exploring urban–rural variations and contributing factors

**DOI:** 10.3389/fmed.2024.1458829

**Published:** 2024-09-17

**Authors:** Xin-He Fang, De-Sheng Song, Nan Jin, Bei Du, Rui-Hua Wei

**Affiliations:** ^1^Tianjin Key Laboratory of Retinal Functions and Diseases, Tianjin Branch of National Clinical Research Center for Ocular Disease, Eye Institute and School of Optometry, Tianjin Medical University Eye Hospital, Tianjin, China; ^2^Ningxia Eye Hospital, People’s Hospital of Ningxia Hui Autonomous Region, Third Clinical Medical College of Ningxia Medical University, Yinchuan, China

**Keywords:** urban–rural differences, prevalence, risk factors, refractive errors, Tianjin, China

## Abstract

**Purpose:**

Refractive errors, particularly myopia, constitute a significant global public health concern, contributing to morbidity and disability. A more comprehensive understanding of the determinants of refractive errors and the differences between urban and rural areas is essential to develop effective preventive measures for youth. This study aimed to compare the prevalence and risk factors of refractive errors among youth in urban and rural Tianjin, China.

**Methods:**

This school-based cross-sectional study was conducted in 2022. Elementary, middle, and high school students aged 6–18 years from both urban and rural areas of Tianjin were included. All participants underwent visual acuity testing and refractive measurement and completed comprehensive questionnaires.

**Results:**

A total of 346,146 participants (176,628 boys) were included in this investigation (50.36% for urban and 49.64% for rural, respectively). Myopia, hyperopia, astigmatism, and anisometropia were present in 56.8, 9.7, 56.64, and 21.3% of urban students, respectively. Similarly, rural students had a prevalence of 57.6, 11.5, 56.48, and 22.0% for the respective conditions. Compared to rural students, after adjusting for age, sex, and other significant variables, urban students were 1.05 times more likely to have myopia (95% CI: 1.03–1.07, *p* < 0.0001), 0.71 times less likely to have hyperopia (95% CI: 0.69–0.73, *p* < 0.0001), and 1.02 times more likely to have astigmatism (95% CI: 0.69–0.73, *p* < 0.0001). There was no significant association between anisometropia and residence (OR: 1.00, 95% CI: 0.98–1.02, *p* = 0.9850). Sociodemographic and physiological factors contribute to the disparities in the prevalence of refractive errors between urban and rural areas. Age, increased near-work activities, and Decreased outdoor time were identified as risk factors for myopia, astigmatism, and anisometropia. Conversely, the absence of a parental history of refractive errors emerged as a protective factor for myopia and astigmatism among students. Lower parental education levels were negatively correlated with the risk of myopia and anisometropia in their children. Specifically, the lower the parental education, the greater the risk of myopia in their offspring. For urban students only, lower parental education was associated with an increased risk of astigmatism.

**Conclusion:**

Crude prevalence estimates May not accurately reflect the true burden of refractive error due to confounding factors such as age and sex. Accounting for these factors revealed that urban students were more likely to have myopia and astigmatism but less likely to have hyperopia compared to their rural counterparts. These disparities highlight the importance of considering geographical variations when implementing strategies for myopia control and prevention.

## 1 Background

The increasing prevalence of refractive errors (REs) has become a major public health concern, posing a significant threat to vision due to the potential for sight-threatening complications like retinal detachment and glaucoma (1, 2). Myopia, the most common RE, is a major contributor to visual impairment globally (3). Recognizing this growing concern, the China National Health Commission (NHC) integrated myopia prevention and control into public health priorities by issuing the “Guidelines on Appropriate Techniques for the Prevention and Control of Childhood and Adolescent Myopia” in 2019. Data from the National Bureau of Disease Prevention and Control’s monitoring program revealed a national prevalence of myopia in Chinese children and adolescents of 51.9% in 2022. This represents a Decrease of 0.7 percentage from 52.6% in 2021 and 1.7 percentage from 53.6% in 2018 (4). Notably, among students diagnosed with myopia, 53.3% had mild myopia, 37.0% had moderate myopia, and 9.7% had high myopia (4).

The pathogenesis of myopia has not yet been fully elucidated, and its development is attributed to a complex interplay of multiple factors. Genetic and environmental factors are now understood to be implicated in the development of myopia, as indicated by epidemiologic and genetic research (5, 6). The natural and social environments undergo rapid change, whereas genes do not undergo significant change between generations. Modifiable environmental risk factors can serve as a critical foundation for the development of preventive interventions. In terms of environmental factors, epidemiologic studies have identified predictors such as education, close work (e.g., reading, viewing television, using cell phones or computers), and outdoor activities that are significantly associated with myopia (7). At the same time, gender is also considered to be one of the reasons affecting the prevalence of myopia (8, 9). Current evidence suggests that REs are significantly influenced by place of residence. For instance, a survey in Dalian of 4,522 urban and rural school-age children and adolescents aged 6–18 years found that (10) the prevalence of myopia and anisometropia was higher in urban students than in rural students, while the prevalence of hyperopia was lower in urban students than in rural students. The prevalence of astigmatism did not differ significantly between urban and rural students after adjusting for student age and gender. This suggests that the prevalence of REs is influenced by a variety of physiological and sociodemographic factors. The Beijing Pediatric Ophthalmology Study discovered a similar correlation between urban residence and myopia prevalence. However, the association was no longer significant after adjusting for factors like age, gender, school type, family income, and parental myopia (11). At present, the prevalence and risk factors of REs in youth in Tianjin, both rural and urban, are unknown. To comprehend the disparities in the prevalence of RE and the risk factors associated with it among school-aged children and adolescents in urban and rural regions of Tianjin, we implemented this school-based cross-sectional sample study.

## 2 Objects and methods

### 2.1 Objects

**Figure 1:**
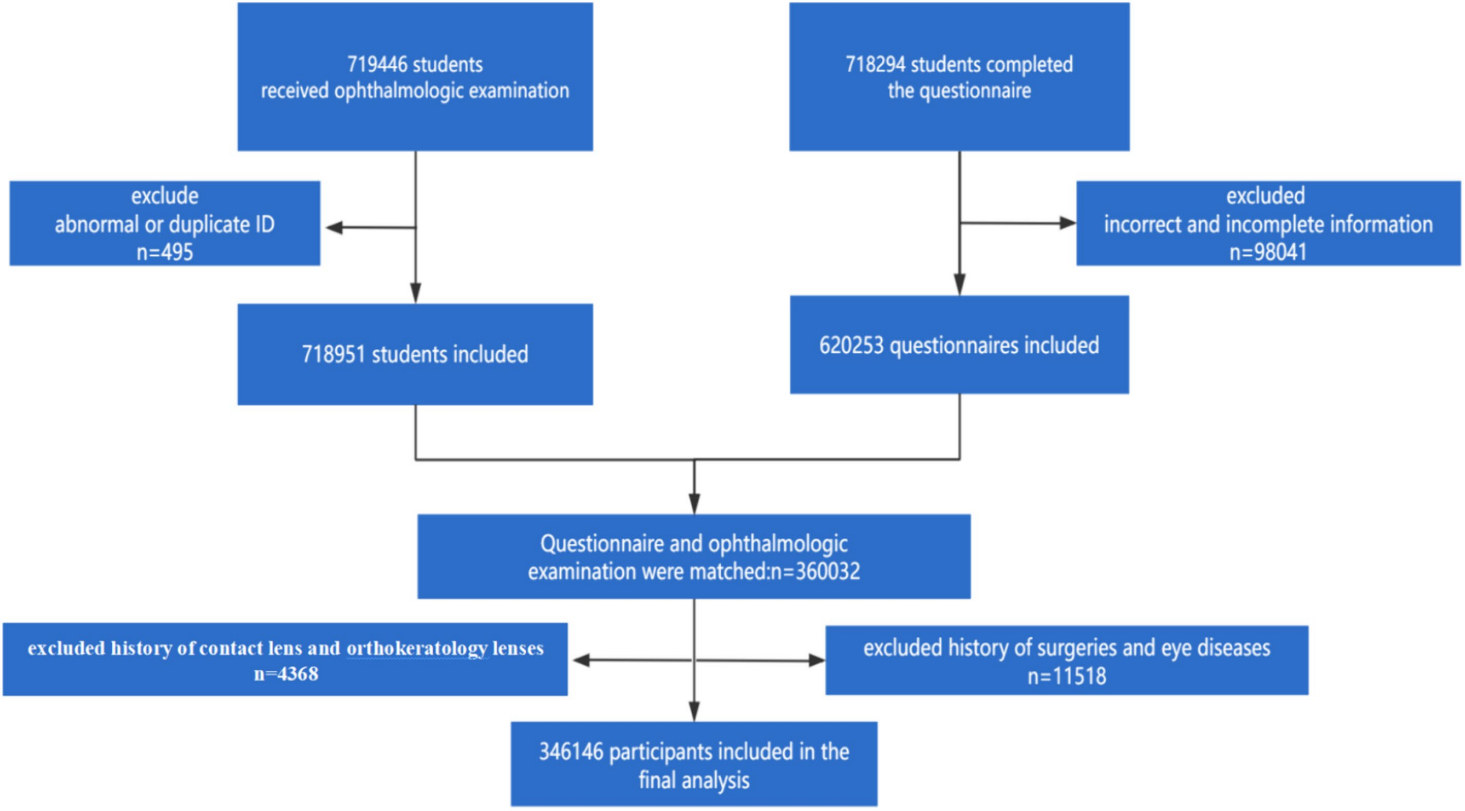
Flow diagram of study participants.

The present study, a school-based cross-sectional study, was conducted in July 2022 in both urban and rural areas of Tianjin, China. The urban areas encompassed six central districts: Heping, Hebei, Hedong, Hexi, Hongqiao, and Nankai. The rural areas comprised five suburban districts: Wuqing, Baodi, Jinghai, Jizhu, and Ninghe. Participants were school-aged children and adolescents aged 6 to 18 years who were enrolled in elementary, middle, and high schools. A cross-sectional design was employed for this study. Exclusion criteria included: (1) participants who had undergone cataract surgery, laser refractive surgery, or were using low-dose atropine; and (2) participants whose parents Declined to sign the informed consent form. A total of 346,146 eligible participants were identified for enrollment in the study. The participant recruitment flowchart is illustrated in Figure 1.

This study was reviewed and approved by the Ethics Committee of Tianjin Medical University Eye Hospital. Written informed consent was obtained from the guardians of all minor children in accordance with the Declaration of Helsinki.

### 2.2 Methods

#### 2.2.1 Questionnaire and data collection

To ensure data quality, class teachers distributed a comprehensive questionnaire to all subjects and their parents. The questionnaire, divided into two sections for students and parents, respectively, gathered detailed information. The student section included: (1) basic sociodemographic details like age, gender, residence (urban/rural), grade level, and eye disease history; (2) near work activities, including daily time spent studying, watching television, and using electronic devices; (3) outdoor activities, encompassing average daily outdoor time and weekly exercise frequency; and (4) dietary intake, querying the frequency of consuming fish, shellfish, fruits, vegetables, sweets, fast food, and carbonated drinks. Students completed this section independently or with guardian assistance for those unable to read or understand it. The parent section focused on parental education background and any history of refractive errors.

#### 2.2.2 Uncorrected visual acuity and refractive measurement

Following the removal of glasses for refractive testing, a standardized visual acuity and refraction assessment was performed by a trained optometrist on all participants. Test results were uploaded in real time via a mobile application. Uncorrected Visual Acuity (UCVA) was measured using a standardized logarithmic visual acuity chart. During right eye testing, a non-contact eye patch was used to occlude the left eye. To ensure proper recovery from dark adaptation, the left eye was assessed at 5–10 s intervals after the right eye examination.

This study utilized the TianleRM-9600 autorefractor (Shanghai, China) to obtain non-cycloplegic refraction measurements. Each eye’s refraction was measured three times, and the average value was calculated. If the difference between any two measurements exceeded 0.50 diopter (D), the subject underwent re-examination. Spherical equivalence (SE) was calculated as the sum of spherical dioptres and half the cylinder dioptres. Myopia was defined as SE ≤ −0.50 D. Further classification of myopia severity followed these criteria: low myopia (−3.00 D ≤ SE < −0.50 D), moderate myopia (−6.00 D ≤ SE < −3.00 D), and high myopia (SE < −6.00 D) as established by the Expert Consensus on Refractive Correction in Children (2017). Hyperopia was defined as SE > +0.5 D in one or both eyes. Hyperopia was further categorized as low (+0.50 D < SE ≤ +3.00 D), moderate (+3.00 D < SE ≤ +5.00 D), and high (SE > +5.00 D) based on the same consensus statement. Astigmatism was defined as the absolute value of the cylinder dioptre (|DC|) ≥ +0.75 D in either eye. The severity of astigmatism was classified as mild (0.75 D ≤ |DC| ≤ 1.00 D), moderate (1.00 D < |DC| ≤ 2.00 D), and severe (|DC| > 2.00 D). Anisometropia was defined as the SE difference between the right and left eye exceeding 1.0 D.

#### 2.2.3 Statistical analysis

All data were analyzed using SAS 9.4 software (SAS Institute Inc., Cary, NC, United States). Subjects were categorized into three age groups (6–11 years, 12–15 years, and 16–18 years) corresponding to the distribution of educational levels across elementary, middle, and high school. Continuous variables were presented as mean ± standard deviation (X ± S), while categorical variables were presented as counts and percentages. Chi-square tests were used to compare categorical variables, and *t*-tests were used to analyze continuous variables. Multifactor logistic regression analysis was employed to assess the correlation between refractive error and other variables within each region. The strength of the association was expressed using the odds ratio (OR) and its corresponding 95% confidence interval (CI). A *p*-value less than 0.05 was considered statistically significant.

## 3 Results

This study included a total of 346,146 school-aged children and adolescents aged 6–18 years, 51.03% for males and 48.97% for females, respectively. Table 1 presents a comparison of sociodemographic characteristics between urban and rural students. Urban students comprised 50.36% (*n* = 174,313) of the sample, with rural students accounting for the remaining 49.64% (*n* = 171,833). Significant differences were observed in terms of gender and age distribution. Urban students had a higher proportion of participants aged 6–11 years (62.0%) compared to rural students (55.5%), while the proportion of students aged 12–15 years was significantly higher in rural areas (34.5%) compared to urban areas (28.4%). Urban students also reported significantly higher levels of parental education and parental refractive error compared to their rural counterparts (*p* < 0.0001). Daily activity patterns differed significantly between the two groups, with urban students engaging in more than 2 h of near-work activities and less than 2 h of outdoor activities per day at a higher rate than rural students (*p* < 0.0001). Urban students further reported higher consumption of whole grains, fish and shrimp, and fresh fruits and vegetables compared to rural students (*p* < 0.0001). However, their diet also included significantly more fried foods, snacks, and carbonated beverages (*p* < 0.0001). Rural students exhibited significantly greater height, weight, and body mass index (BMI) compared to their urban counterparts (*p* < 0.0001). Finally, urban students demonstrated slightly higher absolute refraction values compared to rural students (*p* < 0.0001).

**Table 1:** Distribution and demographic characteristics of the study population.

Characteristic	Overall *N* (%) /Mean ± SD	Urban *N* (%) /Mean ± SD	Rural *N* (%) /Mean ± SD	*p*
Age(years)				<0.0001
6–11	203,475 (58.8)	108,076 (62.0)	95,399 (55.5)	
12–15	108,822 (31.4)	49,539 (28.4)	59,283 (34.5)	
16–18	33,848 (9.8)	16,697 (9.6)	17,151 (10.0)	
Gender				<0.0001
M	176,628 (51.0)	89,555 (51.4)	87,073 (50.7)	
F	169,517 (49.0)	84,757 (48.6)	84,760 (49.3)	
Height (cm)	149.5 ± 17.0	148.4 ± 17.2	150.7 ± 16.7	<0.0001
Weight (cm)	41.9 ± 13.8	40.8 ± 13.8	43.0 ± 13.7	<0.0001
BMI (kg/m2)	18.2 ± 3.3	18.0 ± 3.3	18.5 ± 3.3	<0.0001
Parental refractive error (%)				<0.0001
Myopia	146,331 (42.3)	95,826 (55.0)	50,505 (29.4)	
No Myopia	199,814 (57.7)	78,486 (45.0)	121,328 (70.6)	
Parental education level				<0.0001
Middle school	95,150 (27.5)	12,564 (7.2)	82,586 (48.1)	
Vocational and general high school	123,104 (35.6)	57,713 (33.1)	65,391 (38.1)	
Bachelor	101,195 (29.2)	79,431 (45.6)	2092 (1.2)	
Master	26,696 (7.7)	24,604 (14.1)	2092 (1.2)	
Daily Hours of near-work (%)				<0.0001
<2 h	31,511 (9.1)	13,699 (7.9)	17,812 (10.4)	
≥2 h	314,634 (90.9)	160,613 (92.1)	154,021 (89.6)	
Daily Hours of outdoor activities (%)				<0.0001
<2 h	290,866 (84.0)	158,485 (90.9)	132,381 (77.0)	
≥2 h	55,279 (16.0)	15,827 (9.1)	39,452 (23.0)	
Diet custom				
Intake frequency of whole grains				<0.0001
≤3 times/week	275,875 (79.7)	139,775 (80.2)	136,100 (79.2)	
>3 times/week	70,270 (20.3)	34,537 (19.8)	35,733 (20.8)	
Intake frequency of fish and shrimp				<0.0001
≤3 times/week	312,392 (90.2)	153,799 (88.2)	158,593 (92.3)	
>3 times/week	33,753 (9.8)	20,513 (11.8)	13,240 (7.7)	
Intake frequency of fruit and vegetable				<0.0001
≤3 times/week	66,141 (19.1)	26,249 (15.1)	39,892 (23.2)	
>3 times/week	280,004 (80.9)	148,063 (84.9)	131,941 (76.8)	
Intake frequency of snack				<0.0001
≤3 times/week	268,919 (77.7)	130,773 (75.0)	138,146 (80.4)	
>3 times/week	77,226 (22.3)	43,539 (25.0)	33,687 (19.6)	
Intake frequency of carbonated beverage				<0.0001
≤3 times/week	324,154 (93.6)	160,150 (93.2)	164,004 (94.1)	
>3 times/week	21,991 (6.4)	11,683 (6.8)	10,308 (5.9)	
Spherical equivalence (D)				
OD	−1.6 ± 2.1	−1.7 ± 2.1	−1.6 ± 2.1	<0.0001
OS	−1.5 ± 2.5	−1.6 ± 2.7	−1.5 ± 2.3	<0.0001

Table 2 presents the regional distribution of RE prevalence. Significant variations were observed in RE prevalence between urban and rural students. The prevalence of low and moderate myopia was Marginally lower in the urban population (32.4 and 19.2%, respectively) compared to rural students (33.0 and 19.9%, respectively) (*p* < 0.0001). However, urban students exhibited a higher prevalence of high myopia (5.2%) compared to their rural counterparts (4.7%) (*p* < 0.0001). Rural students had a considerably higher prevalence of hyperopia (11.5%) than urban students (9.7%) (*p* < 0.0001). Within the rural population, the prevalence of low, moderate, and high hyperopia was 10.6, 0.59, and 0.31%, respectively, exceeding that of the urban population (low: 9.03%, moderate: 0.51%, high: 0.16%). The overall prevalence of astigmatism was slightly higher in the urban population (56.64%) compared to rural students (56.48%) (*p* < 0.0001). The distribution of severity within astigmatism categories also differed slightly between the groups. The prevalence of low, moderate, and high astigmatism in the urban population was 26.45, 20.47, and 9.73%, respectively, while the rural population displayed a prevalence of 27.51, 20.35, and 8.62% for low, moderate, and severe astigmatism, respectively. Finally, the prevalence of anisometropia was significantly lower in urban students (21.3%) compared to rural students (22.0%) (*p* < 0.0001).

**Table 2:** Crude prevalence of different refractive errors in urban and rural residents.

Items	Overall % (95% CI)	Urban % (95% CI)	Rural % (95% CI)	*p*
Myopia	57.2% (56.87–57.23%)	56.8% (56.63–57.15%)	57.6% (57.36–57.84%)	<0.0001
Low myopia	32.7% (32.54–32.85%)	32.4% (32.19–32.63%)	33.0% (32.76–33.20%)	
Moderate myopia	19.6% (19.43–19.69%)	19.2% (19.02–19.39%)	19.9% (19.74–20.11%)	
High myopia	4.9% (4.86–5.0%)	5.2% (5.1–5.3%)	4.7% (4.6–4.8%)	
Hyperopia	10.6% (10.51–10.71%)	9.7% (9.56–9.84%)	11.5% (11.38–11.68%)	<0.0001
Low hyperopia	9.86% (9.83–9.88%)	9.03% (9.01–9.05%)	10.6% (10.55–10.64%)	
Moderate hyperopia	0.55% (0.52–0.58%)	0.51% (0.47–0.54%)	0.59% (0.55–0.63%)	
High hyperopia	0.24% (0.22–0.25%)	0.16% (0.14–0.18%)	0.31% (0.28–0.33%)	
Astigmatism	56.56% (56.38–56.74%)	56.64% (56.38–56.90%)	56.48% (56.23–56.73%)	<0.0001
Mild astigmatism	27.00% (26.84–27.16%)	26.45% (26.22–26.68%)	27.51% (27.29–27.73%)	
Moderate astigmatism	20.41% (20.26–20.55%)	20.47% (20.26–20.68%)	20.35% (20.15–20.55%)	
Severe astigmatism	9.15% (9.04–9.25%)	9.73% (9.57–9.88%)	8.62% (8.48–8.76%)	
Anisometropia	21.6% (21.48–21.76%)	21.3% (21.09–21.47%)	22.0% (21.77–22.16%)	<0.0001

Bivariate analysis, as shown in Table 3, identified factors associated with refractive errors and residence region. In both urban and rural students, myopia and hyperopia were significantly correlated with student age, parental education history, time spent on near work activities and outdoor activities, and frequency of consumption of fruits, vegetables, and carbonated beverages (*p* < 0.0001). Interestingly, the frequency of snack intake was not associated with myopia in either urban (*p* = 0.3964) or rural areas (*p* = 0.2426). Similarly, fish and shrimp intake correlated with myopia only in rural students (*p* < 0.0001) but not in urban students (*p* = 0.2134), and it did not correlate with hyperopia (*p* > 0.05). Astigmatism was associated with age, parental history of refractive error, time spent on near-work activities and outdoor activities, and frequency of intake of fruits and vegetables, carbonated beverages, and fish and shrimp in both urban and rural students (*p* < 0.0001). However, parental education was only a significant factor for astigmatism in urban students (*p* < 0.05), not in rural students (*p* > 0.05). Urban students displayed a correlation between snack intake and astigmatism (*p* < 0.05), whereas this association was not observed in rural students (*p* = 0.3932). Finally, significant correlations (*p* < 0.05) were identified between anisometropia in both urban and rural students and their age, time spent on near-work activities and outdoor activities, and frequency of intake of fruits, vegetables, and carbonated beverages. The frequency of snack, fish, and shrimp intake did not correlate with anisometropia in either urban or rural students (*p* > 0.05).

**Table 3:** Bivariate regression results for urban and rural participants.

Characteristic	Myopia		Hyperopia		Astigmatism		Anisometropia	
	Rural OR (95%CI) *p*	Urban OR (95%CI) *p*	Rural OR (95%CI) *p*	Urban OR (95%CI) *p*	Rural OR (95%CI) *p*	Urban OR (95%CI) *p*	Rural OR (95%CI) *p*	Urban OR (95%CI) *p*
Age								
6–11	1	1	1	1	1	1	1	1
12–15	4.51 (4.41,4.62) *p* < 0.0001	4.73 (4.61,4.84) *p* < 0.0001	0.31 (0.30,0.32) *p* < 0.0001	0.33 (0.31,0.34) *p* < 0.0001	1.70 (1.67,1.74) *p* < 0.0001	2.39 (2.34,2.45) *p* < 0.0001	1.88 (1.84,1.93) *p* < 0.0001	2.38 (2.32,2.44) *p* < 0.0001
16–18	10.8 (10.3,11.4) *p* < 0.0001	9.41 (8.98,9.87) *p* < 0.0001	0.17 (0.15,0.18) *p* < 0.0001	0.23 (0.21,0.25) *p* < 0.0001	2.18 (2.10,2.26) *p* < 0.0001	3.09 (2.98,3.21) *p* < 0.0001	2.26 (2.18,2.34) *p* < 0.0001	3.23 (3.11,3.34) *p* < 0.0001
Gender								
M	1	1	1	1	1	1	1	1
F	1.29 (1.27,1.32) *p* < 0.0001	1.29 (1.27,1.32) *p* < 0.0001	0.67 (0.61,0.73) *p* < 0.0001	0.61 (0.55,0.68) *p* < 0.0001	0.88 (0.86,0.90) *p* < 0.0001	0.90 (0.89,0.92) *p* < 0.0001	1.06 (1.04,1.09) *p* < 0.0001	1.09 (1.07,1.12) *p* < 0.0001
Parental refractive error (%)								
Myopia	1	1	1	1	1	1	1	1
No Myopia	0.67 (0.66,0.69) *p* < 0.0001	0.70 (0.69,0.71) *p* < 0.0001	1.34 (1.30,1.39) *p* < 0.0001	1.44 (1.39,1.49) *p* < 0.0001	0.81 (0.79,0.82) *p* < 0.0001	0.88 (0.87,0.90) *p* < 0.0001	0.95 (0.93,0.98) *p* 0.0002	1.02 (1.00,1.04) *p* 0.0970*
Parental education level								
Middle school	1.39 (1.28,1.52) *p* < 0.0001	1.39 (1.28,1.52) *p* < 0.0001	0.62 (0.55,0.70) *p* < 0.0001	0.65 (0.60,0.70) *p* < 0.0001	1.01 (0.92,1.10) *p* 0.6433*	1.19 (1.14,1.25) *p* 0.0028	1.14 (1.02,1.27) *p* < 0.0001	1.18 (1.12,1.24) *p* < 0.0001
Vocational and general high school	1.23 (1.13,1.34) *p* < 0.0001	1.39 (1.35,1.43) *p* < 0.0001	0.74 (0.65,0.83) *p* < 0.0001	0.70 (0.67,0.74) *p* < 0.0001	1.01 (0.93,1.11) *p* 0.8369*	1.31 (1.27,1.35) *p* 0.0001	1.07 (0.96,1.19) *p* 0.7895*	1.18 (1.14,1.23) *p* < 0.0001
Bachelor	1.07 (0.98,1.17) *p* < 0.0001	1.10 (1.07,1.13) *p* < 0.0001	0.94 (0.83,1.06) *p* < 0.0001	0.88 (0.84,0.92) *p* < 0.0001	1.04 (0.95,1.14) *p* 0.1179*	1.10 (1.07,1.13) *p* 0.0001	1.06 (0.95,1.19) *p* 0.8696*	1.04 (1.00,1.08) *p* < 0.0001
Master	1	1	1	1	1	1	1	1
Daily hours of near-work (%)								
<2 h	1	1	1	1	1	1	1	1
≥2 h	1.85 (1.80,1.91) *p* < 0.0001	1.17 (1.15,1.20) *p* < 0.0001	0.63 (0.60,0.65) *p* < 0.0001	0.65 (0.62,0.68) *p* < 0.0001	1.29 (1.25,1.33) *p* < 0.0001	1.44 (1.39,1.49) *p* 0.0001	1.32 (1.27,1.38) *p* < 0.0001	1.53 (1.46,1.61) p < 0.0001
Daily hours of outdoor activities (%)								
<2 h	1	1	1	1	1	1	1	1
≥2 h	0.80 (0.78,0.82) *p* < 0.0001	0.80 (0.78,0.82) *p* < 0.0001	1.11 (1.07,1.15) *p* < 0.0001	1.12 (1.06,1.18) *p* < 0.0001	0.95 (0.93,0.97) *p* 0.0001	0.85 (0.83,0.88) *p* 0.0001	0.93 (0.91,0.96) *p* < 0.0001	0.91 (0.87,0.95) *p* < 0.0001
Diet custom								
Intake frequency of fish and shrimp								
≤3 times/week	1	1	1	1	1	1	1	1
>3 times/week	1.08 (1.05,1.12) *p* < 0.0001	1.02 (0.99, 1.05) *p* 0.2134*	0.96 (0.90,1.01) *p* 0.1262*	0.98 (0.93,1.03) *p* 0.3300*	1.08 (1.04,1.12) *p* 0.0001	1.07 (1.04,1.11) *p* 0.0001	1.02 (0.98,1.07) *p* 0.2772*	1.03 (0.99,1.07) *p* 0.1127*
Intake frequency of fruit and vegetable								
≤3 times/week	1	1	1	1	1	1	1	1
>3 times/week	0.90 (0.88,0.92) *p* < 0.0001	0.80 (0.78,0.82) *p* < 0.0001	1.09 (1.05,1.13) *p* < 0.0001	1.22 (1.16,1.28) *p* < 0.0001	0.94 (0.92,0.96) *p* 0.0001	0.90 (0.87,0.92) *p* 0.0001	0.94 (0.92,0.97) *p* < 0.0001	0.90 (0.88,0.93) *p* < 0.0001
Intake frequency of snack								
≤3 times/week	1	1	1	1	1	1	1	1
>3 times/week	0.99 (0.97,1.01) *p* 0.3964*	1.01 (0.99,1.04) *p* 0.2426*	1.01 (0.97,1.05) *p* 0.6151*	1.00 (0.96,1.04) *p* 0.9697*	1.01 (0.99,1.04) *p* 0.3932*	0.97 (0.95,1.00) *p* 0.0192	0.98 (0.95,1.01) *p* 0.2229*	1.01 (0.98,1.03) *p* 0.5902*
Intake frequency of carbonated beverage								
≤3 times/week	1	1	1	1	1	1	1	1
>3 times/week	1.59 (1.51,1.67) *p* < 0.0001	1.96 (1.85,2.08) *p* < 0.0001	0.67 (0.61,0.73) *p* < 0.0001	0.61 (0.55,0.68) *p* < 0.0001	1.29 (1.23,1.36) *p* 0.0001	1.62 (1.53,1.71) *p* 0.0001	1.22 (1.15,1.29) *p* < 0.0001	1.50 (1.42,1.60) *p* < 0.0001

OR, odds ratio; CI, Confidence Interval; *no statistical differences.

Logistic regression models were employed to analyze the correlations between regions and REs (Table 4). Compared to rural students, urban students exhibited a significantly increased risk of myopia (OR 1.14, 95% CI 1.13–1.16, *p* < 0.0001) and astigmatism (OR 1.07, 95% CI: 1.06–1.09, *p* < 0.0001) in Model 1, after controlling for age and gender. Conversely, urban students were significantly less likely to be hyperopic (OR 0.73, 95% CI: 0.71–0.74, *p* < 0.0001) compared to rural students. Notably, anisometropia did not demonstrate a significant association with residence (OR 1.02, 95% CI: 1.01–1.04, *p* < 0.0001). Model 2 further adjusted for all variables that were significantly different in the bivariate analysis (in addition to age and sex). Compared to rural students, urban students remained significantly more likely to be myopic (OR: 1.05, 95% CI: 1.03–1.07, *p* < 0.0001) and have astigmatism (OR: 1.02, 95% CI: 1.01–1.04, *p* < 0.0001). Additionally, urban students were significantly less likely to be hyperopic (OR: 0.71, 95% CI: 0.69–0.73, *p* < 0.0001). Importantly, the association between anisometropia and residence remained insignificant (OR: 1.00, 95%CI: 0.98–1.02, *p* = 0.9850).

**Table 4:** Multivariate regression results for RE risk differences in urban and rural residents.

	Myopia		Hyperopia		Astigmatism		Anisometropia	
	Rural OR (95%CI) *p*	Urban OR (95%CI) *p*	Rural OR (95%CI) *p*	Urban OR (95%CI) *p*	Rural OR (95%CI) *p*	Urban OR (95%CI) *p*	Rural OR (95%CI) *p*	Urban OR (95%CI) *p*
Model 1	1	1.14(1.13,1.16) *p* < 0.0001	1	0.73(0.71,0.74) *p* < 0.0001	1	1.07(1.06,1.09) *p* < 0.0001	1	1.02(1.01,1.04) 0.0060
Model 2	1	1.05(1.03,1.07) *p* < 0.0001	1	0.71(0.69,0.73) *p* < 0.0001	1	1.02(1.01,1.04) *p* < 0.0097	1	1.00(0.98,1.02) 0.9850*

Model 1: adjusted for age and gender. Model 2: adjusted for age, gender, and any of the variables analyzed significantly in the bivariate analysis. OR, odds ratio; CI, confidence interval. *No statistical differences.

Bivariate and multivariate analyses identified risk factors for REs in both urban and rural students. Age, Decreased time spent outdoors, and increased time spent in close eye use were consistently associated with a higher risk of myopia, astigmatism, and anisometropia. Notably, a lack of parental history of refractive error emerged as a protective factor for both myopia and astigmatism in both urban (myopia: OR 0.70, 95% CI 0.69–0.71; astigmatism: OR 0.88, 95% CI 0.87–0.90) and rural students (myopia: OR 0.67, 95% CI 0.66–0.69; astigmatism: OR 0.81, 95% CI 0.79–0.82). Interestingly, the absence of parental refractive error was a protective factor for anisometropia in rural students (OR 0.95, 95% CI 0.93–0.98) but an independent risk factor for the condition in urban students (OR 1.02, 95% CI 1.00–1.04). Additionally, parental education level was negatively correlated with the risk of myopia and anisometropia in their children. This suggests that lower parental education is associated with a higher risk of myopia in offspring. Furthermore, in urban students, a higher level of parental education was associated with a lower risk of astigmatism, but this association was not observed in rural students (*p* > 0.05).

For hyperopia, the findings differed from myopia and astigmatism. Increased near-eye work, contrary to expectations, emerged as a protective factor in both urban (OR 0.65, 95% CI 0.62–0.68) and rural students (OR 0.63, 95% CI 0.60–0.65). Conversely, exceeding 2 h of daily outdoor activity was associated with a Marginally increased risk of hyperopia in both urban (OR 1.12, 95% CI 1.06–1.18) and rural groups (OR 1.11, 95% CI 1.07–1.15). Interestingly, a lack of parental history of refractive error was associated with a higher risk of hyperopia in both urban (OR 1.44, 95% CI 1.39–1.49) and rural students (OR 1.34, 95% CI 1.30–1.39). Additionally, parental education level exhibited a negative correlation with the risk of hyperopia in offspring. In other words, lower parental education was associated with a higher risk of hyperopia in children.

**Figure 2:**
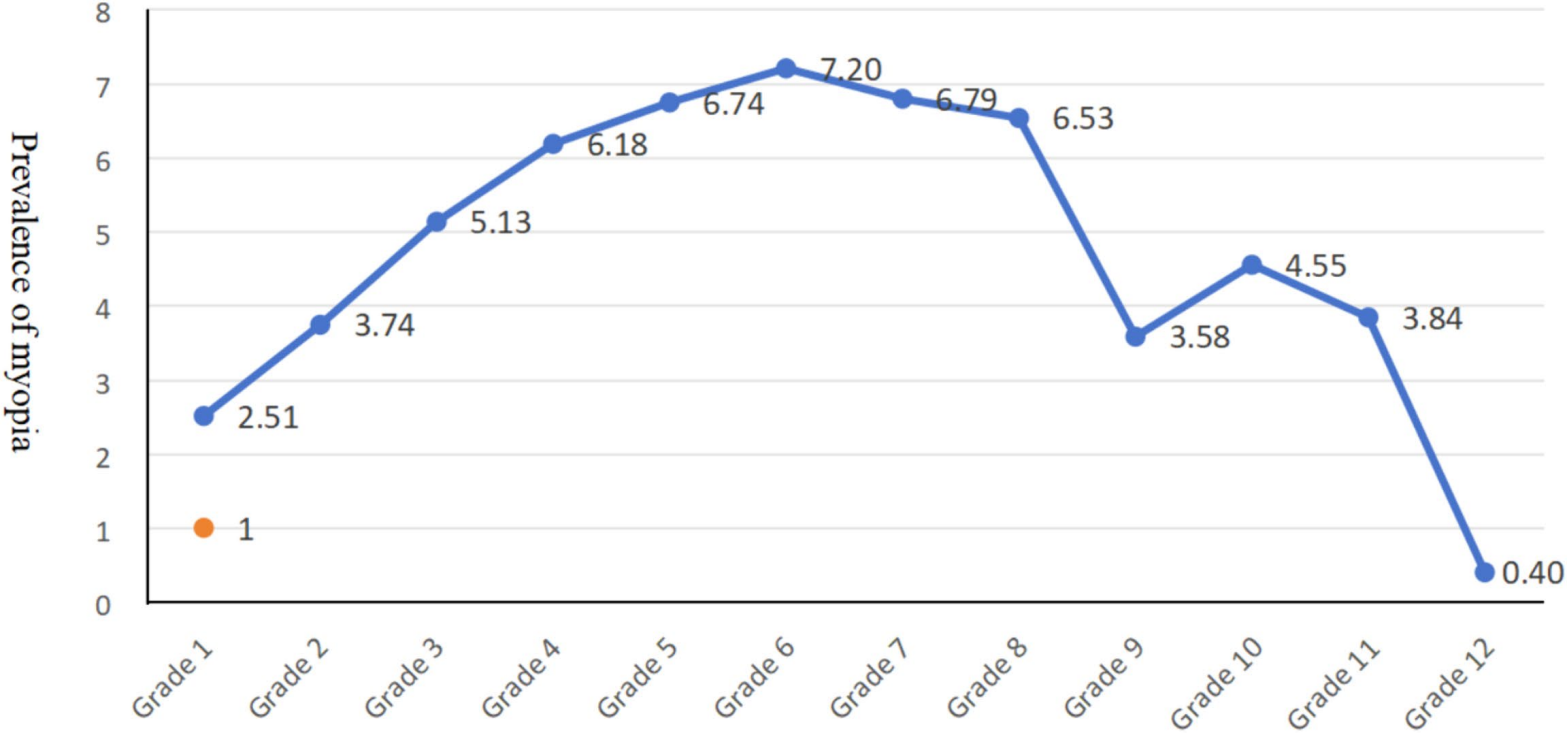
Distribution of myopia trends with grade level in Tianjin, China.

**Figure 3:**
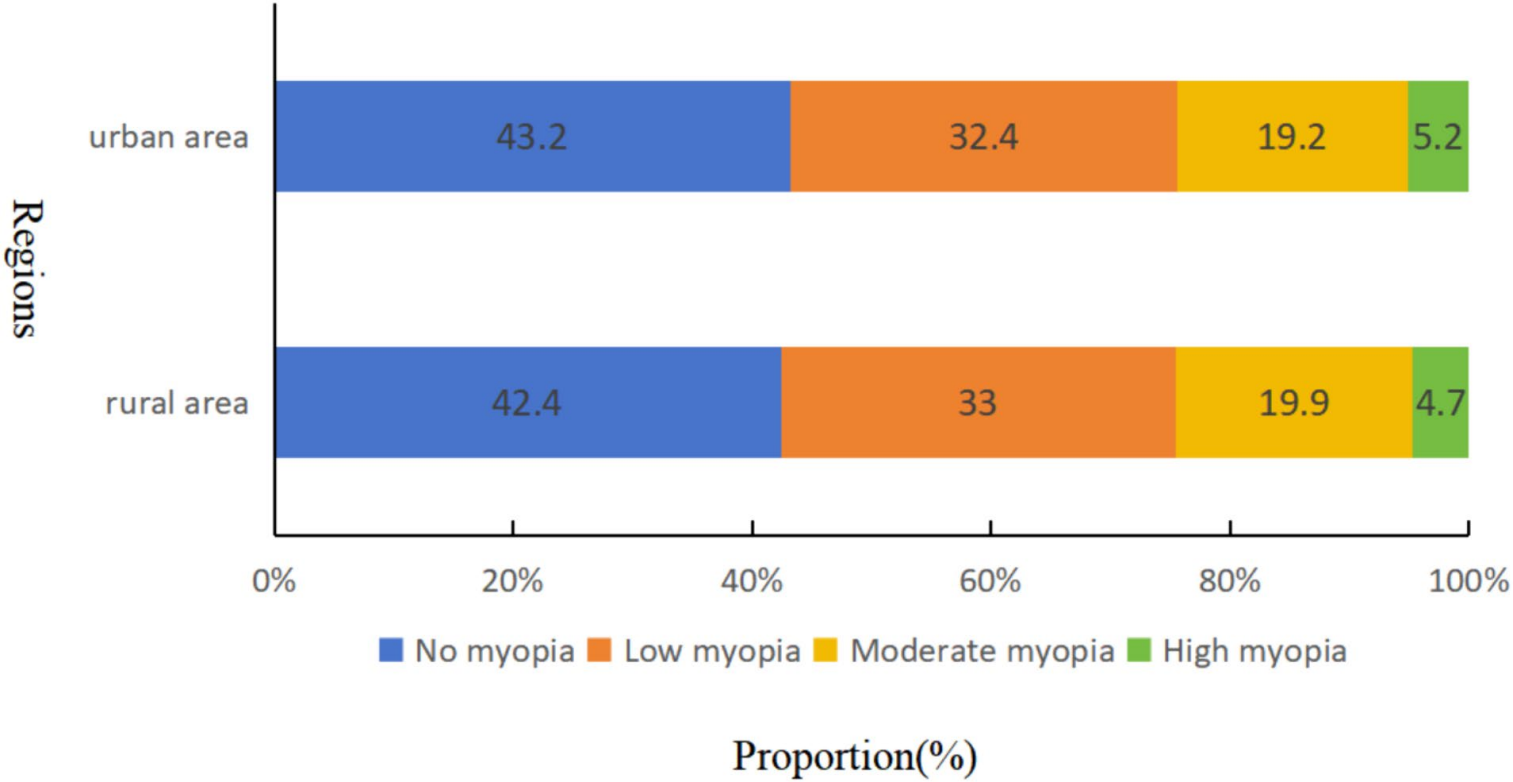
Distribution characteristics of myopia in urban and rural areas of Tianjin, China.

Our findings revealed an age-related trend in myopia prevalence (Figure 2). Myopia prevalence increased more rapidly in elementary and middle school students. Furthermore, our study observed a narrowing gap in myopia prevalence between urban and rural students (Figure 3). Our study found only a 1.05-fold higher prevalence of myopia in urban students compared to rural student.

## 4 Discussion

This study employed a school-based cross-sectional analysis to investigate the prevalence of refractive errors and their associated risk factors among school children and adolescents of varying ages in urban and rural areas of Tianjin, China. We recognized that crude prevalence rates, without adjustment for confounding factors like gender and age, might not accurately reflect the true prevalence across different regions. Our analysis, after adjusting for these influential variables, revealed a higher prevalence and risk of myopia and astigmatism in urban students compared to their rural counterparts. Conversely, the prevalence and risk of hyperopia were lower in urban areas. Interestingly, no significant difference was observed in the prevalence and risk of anisometropia between urban and rural students. Finally, the observed disparity in RE prevalence between urban and rural areas May be attributed to various factors, including time spent in near-work activities, time spent outdoors, parental history of refractive error, and parental educational level.

**Figure 4:**
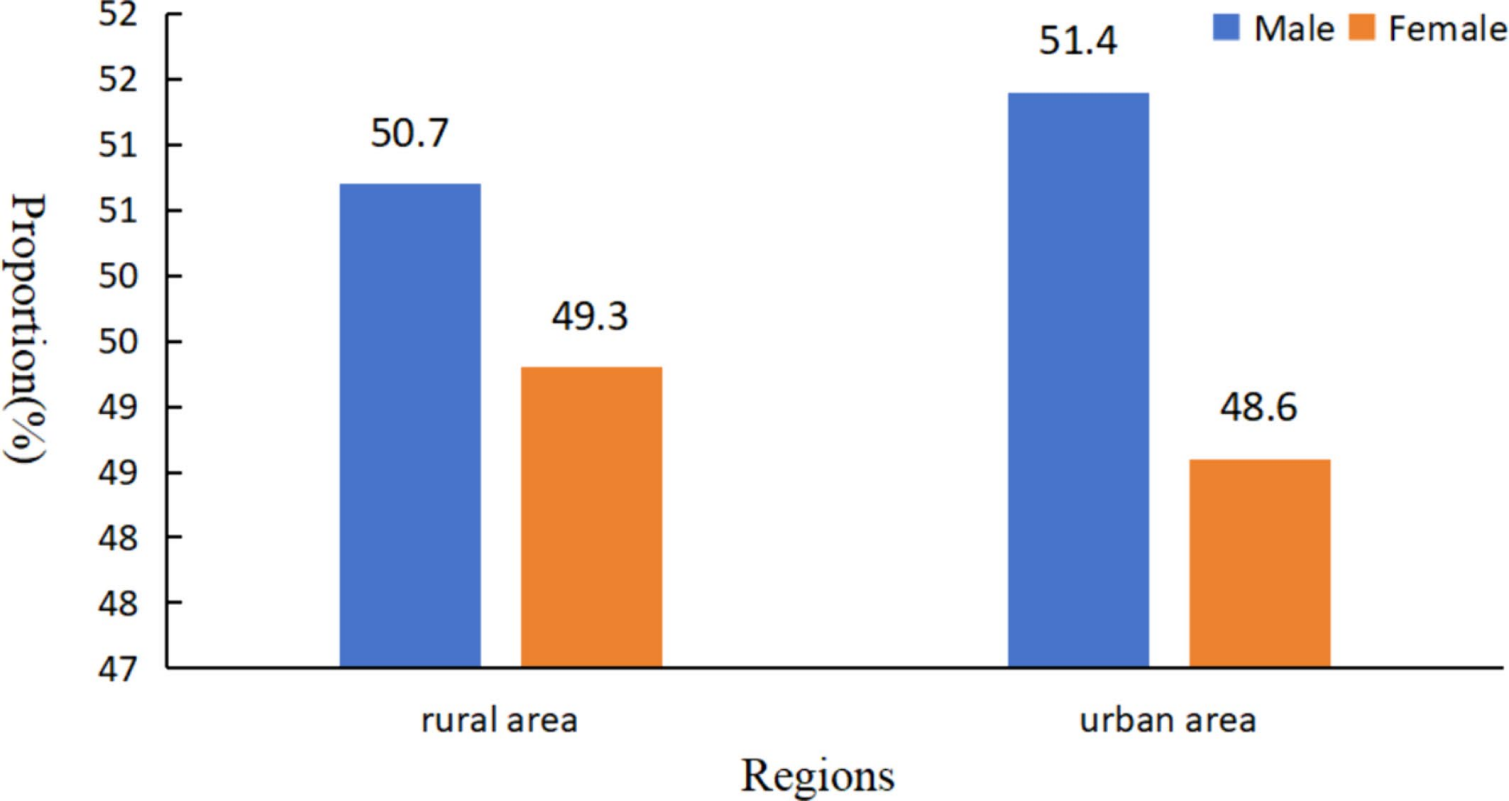
Ratio of male-to-female student composition in urban and rural areas of Tianjin, China.

In 2021, Tang et al. conducted a systematic review and meta-analysis, highlighting regional variations in RE prevalence among Chinese children and adolescents (12). Consistent with our findings, several studies reported a higher prevalence of myopia in urban students compared to rural students across various regions. For instance, an epidemiological study in Youyang County, Chongqing, documented a substantially higher prevalence of myopia in urban adolescents (77.9%) compared to their rural counterparts (51.6%) (13). He et al. observed a rising prevalence of myopia in 12-, 13-, 14-, and 15-year-olds in urban areas of southern China (49.7, 57.4, 65.5, and 78.4%, respectively). In rural areas of southern China, the prevalence of myopia among 13-, 14-, 15-, 16-, and 17-year-olds was 36.8, 38.8, 43.0, 46.8, and 53.9%, respectively (14). Additionally, the prevalence of high myopia was significantly higher in urban adolescents (4.2%) compared to rural adolescents (0.5%) (14). Similar trends were reported in Mianyang, Sichuan Province (urban: 16.28%, rural: 14.37%) (15) and Xingyi, Guizhou Province (urban: 25.4%, rural: 19.6%) (16). Interestingly, our study revealed a Marginally higher crude prevalence of myopia in rural areas compared to urban areas. However, this difference can likely be attributed to the gender composition of the study population. Figure 4 demonstrates a slightly higher proportion of girls in rural areas (49.3%) compared to urban areas (48.6%). Conversely, the percentage of boys in urban areas was 51.4%, which was higher than that of boys in rural areas (50.7%). Previous research suggests that girls are more susceptible to myopia at earlier ages due to their faster pubertal development and emmetropization processes (17). Therefore, the observed discrepancy in crude prevalence May reflect the higher proportion of females in the rural samples.

Urban students were 1.05 times more likely to be myopic than rural students after adjusting for relevant factors identified in the bivariate analysis (age, sex, etc.). This statistically significant association strengthens the validity of our control variables. Consistent with previous reports on primary school students in Beijing (18), we observed that myopia in both urban and rural students was associated with age, Decreased time spent outdoors, and increased time spent on near-work activities. This urban–rural disparity might be explained by several factors: the dense urban environment with tall buildings, adolescents’ frequent exposure to electronic devices, and potentially more demanding schoolwork. Interestingly, the risk of myopia in children was negatively correlated with the level of parental education, suggesting a possible link between parental awareness and emphasis on refractive health. Yi Tang’s 2021 report (12) documented a national prevalence of high myopia (2.8%) in Chinese children and adolescents, with a slightly higher prevalence in urban areas (5.2%) compared to rural areas (4.7%). Notably, the past 3 years of home quarantine and online learning have likely exacerbated this trend, potentially accelerating the onset and progression of high myopia. For many years, extensive research has linked the development of myopia to increase near-work activities. Epidemiological studies consistently report a significant rise in myopia risk with activities like reading closer than 30 cm or for extended durations exceeding 30 min (19). Conversely, increased outdoor time has been established as a crucial protective factor against myopia (14). Clinical trials have demonstrated a dose-dependent effect of outdoor exposure on myopia prevention (20). Animal models further elucidate the mechanisms by which bright outdoor light safeguards against myopia development (21). Rural environments typically offer both a wider field of view and greater light intensity compared to urban areas. This difference in light exposure might be one of the key exogenous environmental factors influencing myopia prevalence.

Our findings revealed an age-related trend in myopia prevalence. Myopia prevalence increased more rapidly in elementary and middle school students, likely due to the ongoing changes in refractive state. As children age, their refractive state typically progresses from hyperopia toward emmetropization and then potentially toward myopia in adulthood (22). Furthermore, our study observed a narrowing gap in myopia prevalence between urban and rural students. This trend might be attributed to the evolving visual environment in rural areas. Increased screen time and Decreased outdoor activity among rural children and adolescents, coupled with potentially lower health literacy and limited access to healthcare compared to urban areas, May be contributing factors. These changes in rural lifestyles could explain why the present study found only a 1.05-fold higher prevalence of myopia in urban students compared to rural students.

Our study found a higher prevalence of hyperopia in rural students (11.5%) compared to urban students (9.7%), aligning with findings from an Indian study (23). This suggests a potential association between rural residence and hyperopia. Environmental factors are thought to play a role, although the exact mechanisms remain under investigation. Interestingly, age and increased near-work activity emerged as protective factors for hyperopia in our study. This is in line with research conducted in Dalian, China, which reported a similar trend for both urban and rural students (10). The overall prevalence of hyperopia (10.6%) was high in this study, and regression analysis of hyperopia similarly found an increasing trend in China. This was attributed to the vigorous reform of physical education in China, which encourages children’s outdoor activities (24).

In this study, astigmatism was defined as the absolute value of the cylindrical power (|DC|) in either eye being ≥ +0.75 D. We observed a prevalence of astigmatism in Tianjin of 56.56%, with a statistically significant difference between urban (56.64%) and rural areas (56.48%). This finding contrasts with the national estimate reported by Yi Tang’s 2021 meta-analysis (16.5%) (12). Astigmatism prevalence in Chinese children and adolescents varied significantly by region. In Jinan City, a study found a prevalence of 48.8% among students aged 6–18 years (25). Lin Haishuang et al. investigated the 5-14-year-old population in Wenzhou, employing cycloplegic refraction with a criterion of |DC| ≥ 0.50 D, resulting in a detection rate of 59.4% for astigmatism (26). Conversely, researchers in Taiwan utilized a stricter criterion of|DC| ≥ 1.00 D for astigmatism screening, leading to a lower prevalence of 32.9% (27). These variations highlight the significant impact of differing diagnostic criteria (|DC| ≥ 0.50 D, 0.75 D, and 1.00 D) and the cycloplegic refraction state at the time of examination on the reported prevalence of astigmatism.

The current investigation demonstrated that the prevalence of astigmatism among pupils between the ages of six and 18 in Tianjin increased as they aged. This aligns with a 2011 study by Zhang et al. on primary and secondary school students in Deqing, Zhejiang Province, which also observed an age-related increase in astigmatism (28). Notably, the onset and development of myopia are closely linked to astigmatism. In our study, the prevalence of astigmatism was higher in urban areas than in rural areas. This finding is consistent with the observed higher prevalence of myopia in urban students. Age, increased near-eye use, and Decreased outdoor activities emerged as risk factors for astigmatism in both urban and rural students. This trend is likely related to the increased near-work activities and Decreased outdoor exposure commonly observed among adolescents in urban environments. Interestingly, our study found a lower risk of astigmatism in girls compared to boys in both urban and rural areas. This aligns with previous research. In this respect, a study in Jinan City reported a lower prevalence of astigmatism in girls (47.4%) compared to boys (50.0%) among 6-18-year-olds (25). Similarly, research in the Dalian area documented a lower risk of astigmatism in female urban students aged 6–18 years (10). However, a Singaporean study on children aged 7–9 found a more pronounced progression of astigmatism in girls compared to boys (29).

Despite a large sample size encompassing all school-age children and adolescents in urban and rural Tianjin, our study has limitations. First, the use of non-cycloplegic refraction for refractive data collection May introduce errors. Recent myopia studies on children increasingly advocate for the use of spherical equivalence in conjunction with uncorrected visual acuity for diagnosis, which is the approach employed here. Second, the cross-sectional design precludes longitudinal analyses. Future efforts will focus on monitoring visual acuity in children and adolescents, examining the regression of refractive error with age progression, and investigating related risk factors in both urban and rural areas. Finally, the inclusion of a self-reported questionnaire component introduces the potential for bias during data collection.

## 5 Conclusion

This study investigated the prevalence of refractive errors, including myopia, hyperopia, astigmatism, and anisometropia, among children and adolescents aged 6–18 years in Tianjin, China. We found a significantly higher prevalence of myopia and astigmatism in urban students compared to their rural counterparts, after adjusting for confounding factors. Conversely, hyperopia prevalence was lower among rural students. Interestingly, no significant association was observed between anisometropia and residential areas. Multiple logistic regression analysis was employed to identify risk factors for REs and assess potential disparities between urban and rural participants. In conclusion, the findings from this study can provide valuable insights for developing effective prevention strategies for REs in school-age children and adolescents across urban and rural settings. The results also emphasize the need for tailored myopia prevention and control programs considering the unique characteristics of different regions.

## Data Availability

The raw data supporting the conclusions of this article will be made available by the authors, without undue reservation.
